# Comparison of three fixation methods in paediatric metaphyseal-diaphysis junction fracture of the distal radius: a retrospective study in two centres

**DOI:** 10.3389/fped.2023.1244704

**Published:** 2023-08-24

**Authors:** Jiang Jianyi, Liu Chaoyu, Meng Lian, Meng Ge, Ma Hailong, Sun Jun, Jia Guoqiang

**Affiliations:** ^1^ Department of Orthopedics, Children’s Hospital of Anhui Medical University, Hefei, China; ^2^ The People's Hospital of Fuyang of Anhui Medical University, Fuyang, China

**Keywords:** DRMDJ, radius, ESIN, precision shaping, retrograde, children level: III

## Abstract

**Background:**

The distal radial metaphyseal-diaphysis junction fractures (DRMDJ) have various treatment methods and are easily lead to complications. This study aims to compare the anterograde elastic stable intramedullary nailing (ESIN-A), retrograde K-wire fixation (KW-R), and retrograde precision-shaping elastic intramedullary nailing (ESIN-RPS) for the treatment of pediatric DRMDJ fractures.

**Materials and methods:**

A total of 113 patients with DRMDJ fractures (36 in the ESIN-A group, 52 in the KW-R group, and 25 in the ESIN-RPS group) from two centres were retrospectively analysed. Perioperative operation time, intraoperative bleeding, fluoroscopy times, alignment rate and angulation on radiography were compared among the three groups. Forearm rotation, healing, wrist function, and complications were compared at the last follow-up.

**Results:**

The mean operation times of the three groups were as follows: KW-R (72 ± 13 min) > ESIN-A (65 ± 18 min) > ESIN-RPS (52 ± 11 min), with a significant difference (*P* < 0.01). The incision length and intraoperative blood loss of ESIN-A (1.8 ± 0.2 cm; 8.3 ± 3.7 ml) were significantly higher than ESIN-RPS (1.4 ± 0.8 cm; 5.5 ± 2.7 ml) (*P* < 0.05), respectively. The postoperative alignment rate on the anteroposterior (AP) and the lateral plane of ESIN-RPS (93.1 ± 4.4%; 95.01 ± 2.8%) was significantly greater than that of KW-R (82.1 ± 6.8%; 88.5 ± 4.5%) and ESIN-A (79.2 ± 5.2%; 83.2 ± 2.5%) (*P* < 0.01). The residual angulation of ESIN-RPS (3.3 ± 1.2°; 2.9 ± 0.8°) was significantly greater than that for ESIN-A (5.1 ± 1.7°; 4.9 ± 2.1°) and KW-R (6.6 ± 2.8°; 7.5 ± 1.6°) (*P* < 0.05). The excellent and good ratio of ESIN-RPS (95.8%) was significantly higher than that of ESIN-A (86.5%) and KW-R (86.1%) according to the Gartland-Werley standard. There was a significant difference in delayed union between the KW-R and ESIN-A (*P* < 0.05). Additionally, there were two cases of radial nerve injury in the ESIN-A group, one case of tendon rupture in the ESIN-RPS group, and one case of tendon rupture in the KW-R group. The ESIN-RPS group had significantly fewer complications than the KW-R group (*P* < 0.05). The ESIN-A group also had significantly fewer complications than the KW-R group (*P* < 0.05).

**Conclusion:**

Compared with ESIN-A and KW-R, ESIN-RPS has the advantages of a shorter operation time, less intraoperative blood loss, less radiation, better alignment, and fewer complications. The ESIN-RPS was suggested as an optimal choice for paediatric DRMDJ fractures.

## 1. Introduction

Fracture of the distal radial metaphyseal-diaphysis junction (DRMDJ) is identified by Lieber et al. in 2010 and characterized by specific anatomy: (1) the place includes part of the tendon muscle migration and lacks significant muscle attachment on the bone surface; (2) there are fewer vascular perforations than the metaphyseal or shaft portion (1); and (3) the proximal medullary cavity gradually expands toward the distal end (2–4). In terms of treatment, DRMDJ fractures with good post-reduction alignment and stability, as well as those in younger patients, may be treated conservatively. Patients with instability, poor alignment after reduction, and re-displacement after repeated manipulation should be treated surgically, especially older patients with lower remodeling potential (5–11).

Surgical treatment methods for DRMDJ fractures include closed reduction with retrograde K-wires (KW-R), anterograde elastic stable intramedullary nail fixation (ESIN-A), plate fixation, retrograde elastic stable intramedullary nail fixation, and external fixator fixation (3–7, 10–12). Each method possesses advantages and disadvantages. In this study, we propose a new method of retrograde precision shaping ESIN (ESIN-RPS) to treat DRMDJ fractures and compare the preliminary outcomes with ESIN-A and KW-R groups.

## 2. Patients and methods

### 2.1. Patients

A total of 168 patients with DRMDJ fractures who were admitted to Children's Hospital of Anhui Medical University or People's Hospital of Fuyang City between January 2016 and June 2022 were retrospectively analysed. Patients were divided into three groups according to treatment: KW-R, ESIN-A, and ESIN-RPS groups.

The inclusion criteria were as follows: (1) age 5–14 years; (2) diagnosis of DRMDJ fracture with closed reduction failure, unstable, residual angle ≥20°, shortening ≥1 cm, 100% displacement; and (3) follow up >6 months. The exclusion criteria were as follows: (1) having a displaced ulnar fracture requiring surgical treatment; (2) having multiple fractures of the ipsilateral limbs; (3) having open fractures; (4) having iterative fractures; and (5) bone diseases such as neurofibromatosis or osteogenesis imperfecta.

This study was approved by the Institutional Review Board of the Children's Hospital of Anhui Medical University (Approval number: EYLL-2019-035) and the People's Hospital of Fuyang City (Approval number: FYRMH-LL-20200190). It was performed in accordance with the tenets of the Declaration of Helsinki. Consent was obtained from the patients or their guardians.

Fourteen patients in the ESIN-RPS group, nineteen in the ESIN-A group, and twenty-three in the KW-R group were excluded.

### 2.2. Surgical techniques

**Figure 1:**
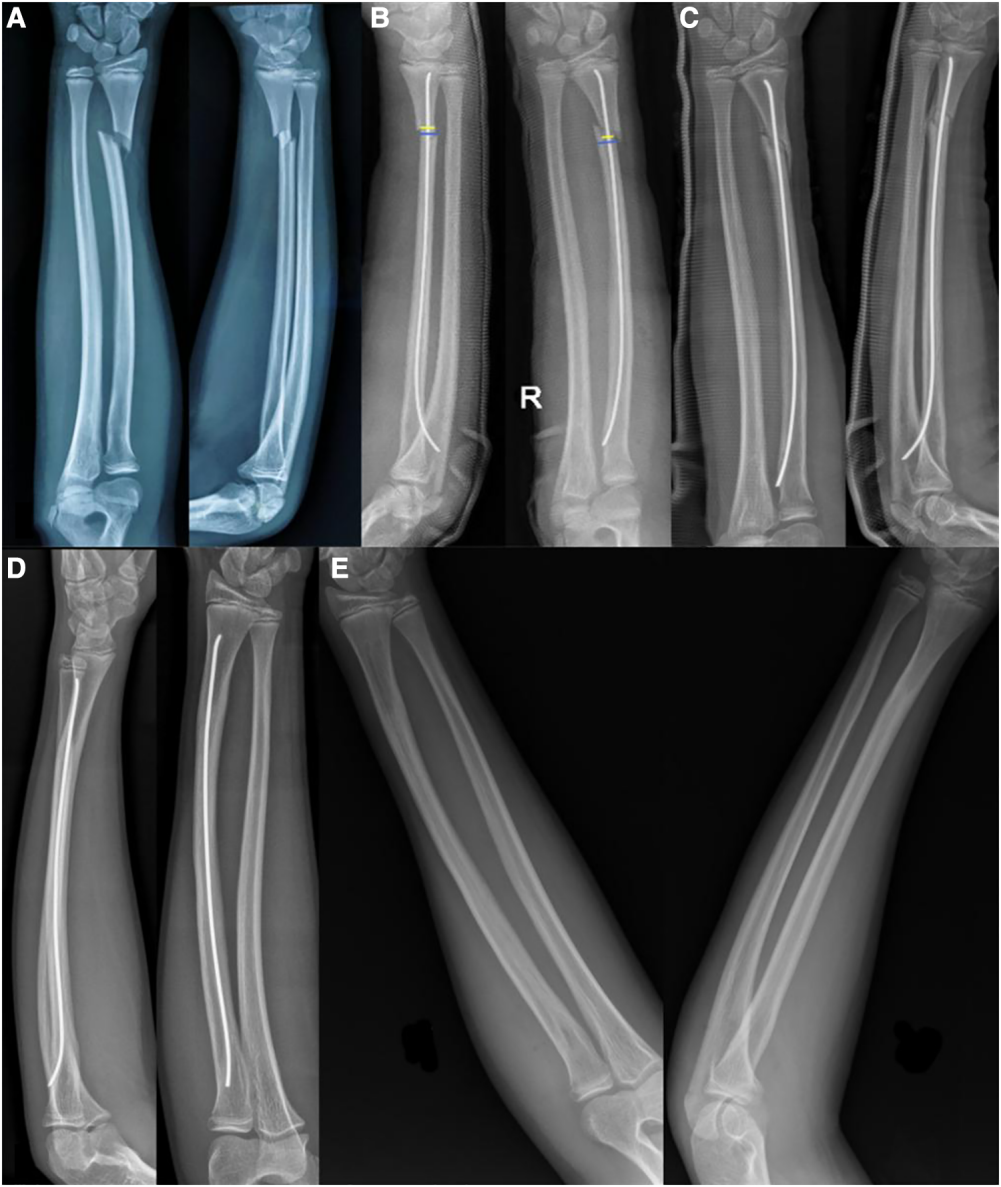
(**A**) An 11-year-old boy with obvious displacement of a right DRMDJ fracture on AP and lateral x-ray films; (**B**) after ESIN-A fixation, the alignment of the fracture was not good and showed residual radial displacement (yellow line was the apposition length of both fragments, blue line was the length of fracture line, and yellow/blue line was the alignment rate). (**C**) Two months after the operation, callus formation was showed, and residual lateral displacement and dorsal angulation were observed. (**D**) One-and-a-half years after the operation, the fracture was completely healed, and the residual dorsal angulation still exists. (**E**) The ESIN was removed, and the fracture completely healed.

**Figure 2:**
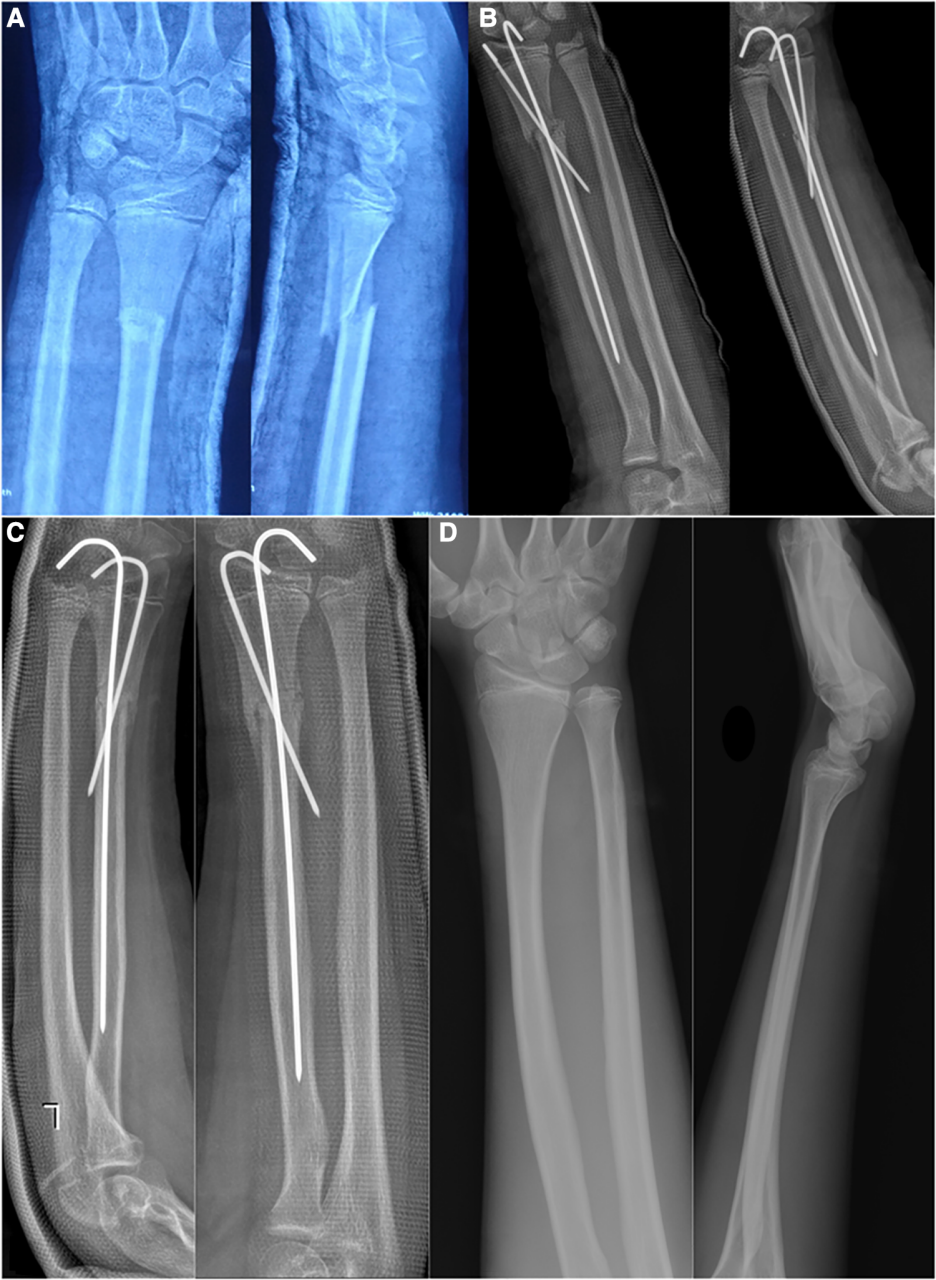
An 11-year-old boy with left DRMDJ fracture. (**A**) Obvious displacement of DRMDJ fracture on AP and lateral x-ray films. (**B**). The x-ray film was rechecked immediately after the operation. There was a residual lateral displacement and dorsal angulation. (**C**) At 6 weeks post-operative, much of the callus formation and the K-wires were removed. (**D**) Three years after the operation the fracture was completely healed on radiography.

**Figure 3:**
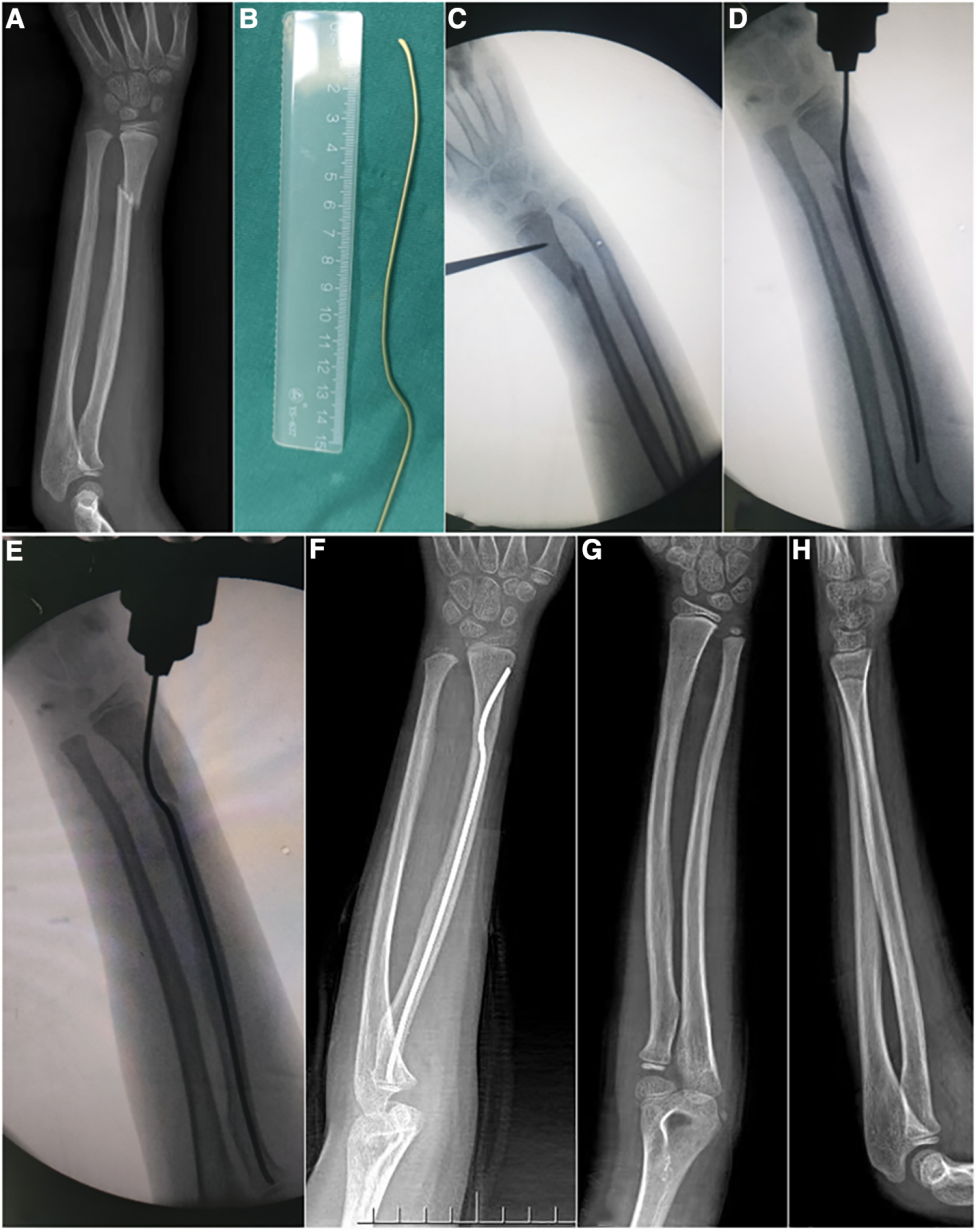
A 7-year-old child with DRMDJ fracture of the left radius. (**A**) x-ray film shows DRMDJ fracture of left radius; (**B**) according to the preoperative x-ray measurement, a precisely shaped ESIN should be pre-bended; (**C**) intraoperative positioning of the insertion point; (**D**) the displacement of the broken end is aggravated by the insertion of the unbending ESIN; (**E**) precise shaping ESIN is placed, and radial displacement disappears; (**F**) three months after the operation, the fracture had healed well; (**G,H**) after removing the elastic intramedullary nail 6 months after the operation, the radius was completely healed.

ESIN-A and KW-R were performed using classic traditionally reported techniques (7, 13); the procedure and follow-up were showed in Figures 1, 2. ESIN-RPS was a novel technique performed by two attending surgeons as follows: (1) The incision length was approximately 1.5 cm in the distal radius and the entry point was determined using fluoroscopy around the classical Lister node; (2) the precision-shape ESIN was then inserted through the point and placed proximal to the physeal plate by 0.5 cm–1 cm; (3) closed reduction of the fracture was performed to allow for better alignment. The surgeon maintained the fracture side with one hand and held the ESIN handle with the other hand, and then slowly inserted the nail until the proximal prebending vertex located on the diaphyseal fracture side, and the distal vertex of the nail prebending was located at the distal end of the fracture line; (4) The quality of reduction was assessed using fluoroscopy, and the tail of the ESIN was then cut and burned on the surface of the deep fascia. The entire procedure is illustrated in Figure 3. A short-arm plaster was used at 90° flexion of the elbow joint.

### 2.3. Evaluation of peri-operative and follow-up outcomes

Operation time, fluoroscopy times, bleeding loss, and fracture alignment rate were recorded. The anteroposterior (AP) and lateral fracture alignment were calculated as follows: (actual contact surface length of two fragments/length of fracture line) * 100% on x-ray films. The plaster was removed 4 weeks postoperatively and active functional exercise was advised in the ESIN-A and ESIN-RPS groups. The plaster and K-wires were removed of the KW-R group according to fracture healing 4 to 8 weeks after surgery. Six months after the fracture healed, the ESIN was removed. At the last follow-up, wrist function was evaluated using the Gartland-Werley criteria, and forearm rotation, infection, and radial nerve injury were evaluated (14).

### 2.4. Statistical analysis

Statistical analysis was performed using IBM SPSS software (version 23.0; IBM Corp., Armonk, NY, USA). Data were presented as mean ± standard deviation. Continuous variables were analysed using Student's *t*-test. Categorical variables were analysed using the *χ*^2^ test or Fisher's exact test. Statistical significance was set as *P*-value <0.05.

## 3. Results

### 3.1. Paitents

In total, 113 patients were retrospectively analysed, including 64 males and 49 females. The mean age was 8.4 years, and the mean time from injury to surgery was 3 days. There were 51 cases on the left side and 62 cases on the right; 20 cases had simple radius fracture, and 93 cases combined with greenstick ulnar fracture. Thirty-six cases were included in the ESIN-A group, fifty-two in the KW-R group, and twenty-five in the ESIN-RPS group.

### 3.2. Perioperative data

The descriptive data of the 113 patients included in the study are summarised in Table 1. The mean operation time was 71.72 ± 12.47 min in the KW-R group, 65.13 ± 18.26 min in the ESIN-A group, and 51.74 ± 11.21 min in the ESIN-RPS group with a statistical difference between the three groups (*P* < 0.05). The mean length of incision and mean intraoperative blood loss were 1.82 ± 0.24 cm and 8.34 ± 3.71 ml in the ESIN-A group, and 1.42 ± 0.83 cm and 5.54 ± 2.73 ml in the ESIN-RPS group. These results were significantly different (*P* < 0.05). The mean times of fluoroscopy were 14.29 ± 3.48 times in the KW-R group, 8.91 ± 4.14 times in the ESIN-A group, and 6.29 ± 2.54 times in the ESIN-RPS group, with significant differences between the three groups (*P* < 0.05). The KW-R group received the highest radiation exposure. The costs of hospitalisation in the ESIN-A group were 14.6 ± 6.8 thousand (RMB), 14.1 ± 1.4 thousand in the ESIN-RPS group, and 11.1 ± 3.6 thousand in the KW-R group with statistical differences (*P* < 0.05). There were no significant differences in the days of hospitalisation amongst the three groups. The alignment rate on AP and lateral radiography postoperatively were 93.1 ± 4.43% and 95.06 ± 2.82% in the ESIN-RPS group, 82.1 ± 6.82%, and 88.52 ± 4.54% in the KW-R group, and 79.17 ± 5.21 and 83.17 ± 2.53% in the ESIN-A group. There were significant differences between the three groups (*P* < 0.05), and the alignment rate in the ESIN-A group was the worst. The AP and lateral angulation on radiography postoperatively were 3.27 ± 1.21° and 2.92 ± 0.78 in the ESIN-RPS group, 5.12 ± 1.68° and 4.89 ± 2.08° in the ESIN-A group, and 6.62 ± 2.84° and 7.53 ± 1.58° in the KW-R group, there were significant differences between these groups (*P* < 0.05).

**Table 1:** Comparison of the perioperative data in three groups.

	ESIN-RPS $\left(\bar{x}\pm s\right)$	KW-R $\left(\bar{x}\pm s\right)$	ESIN-A $\left(\bar{x}\pm s\right)$	*P*_1_/*t*_1_	*P*_2_/*t*_2_	*P*_3_/*t*_3_
Sample (*n*)	25	52	36	/	/	/
Mean age (year)	8.4 ± 2.7	8.8 ± 3.6	7.9 ± 1.7	0.618/0.439	0.132/1.52	0.431/0.796
Surgical time (min)	51.7 ± 11.2	71.7 ± 12.5	65.1 ± 18.3	0.001/6.591	0.002/3.152	0.047/2.013
Incision length (cm)	1.4 ± 0.8	/	1.8 ± 0.2	/	0.008/2.730	/
Blood lost (ml)	6.5 ± 2.7	6.3 ± 1.6	8.3 ± 3.7	0.149/0.454	0.002/3.116	0.001/3.540
Fluoroscopies (times)	6.3 ± 2.5	14.3 ± 3.4	8.9 ± 4.1	0.001/9.903	0.008/2.720	0.001/9.806
Cost (thousand, RMB)	1.4 ± 0.1	1.1 ± 0.4	1.5 ± 0.7	0.001/3.857	0.729/0.346	0.002/3.135
Hospitalisation (days)	4.1 ± 2.3	4.3 ± 1.4	4.8 ± 1.5	0.794/0.260	0.851/0.311	0.949/0.063
Post-operation						
Alignment on AP (%)	93.1 ± 4.4	82.1 ± 6.3	79.2 ± 5.2	0.001/7.712	0.001/10.688	0.028/2.232
Alignment on lateral (%)	95.1 ± 2.3	88.5 ± 4.5	83.2 ± 2.6	0.001/7.413	0.001/16.835	0.001/6.407
Angulation on AP (°)	3.3 ± 1.2	6.6 ± 2.9	5.1 ± 1.7	0.001/5.427	0.001/4.503	0.005/2.840
Angulation on lateral (°)	2.9 ± 0.8	7.5 ± 1.6	4.4 ± 2.1	0.001/13.260	0.001/4.121	0.001/6.763

Where *P* is the statistical probability, *t* is the statistical quantity, *P*_1_/*t*_1_ is ESIN-RP vs. KW-R, *P*_2_/*t*_2_ is ESIN-RP vs. ESIN-A, and *P*_3_/*t*_3_ is KW-R vs. ESIN-A.

### 3.3. Radiographic and functional outcomes

The clinical radiographic and functional outcomes of the 113 patients are summarized in Table 2. The follow-up interview was done via telephone, WeChat, and outpatient clinics. The mean time of follow-up in the three groups was 38.57 ± 12.62 months in the KW-R group, 19.26 ± 9.85 months in the ESIN-A group, and 11.38 ± 2.64 months in the ESIN-RPS group, with significant differences between the three groups (*P* < 0.05). At the last follow-up, there was one case of thumb extension limitation in the ESIN-RPS, one in the KW-R groups, and two cases in the ESIN-A group. There was no significant difference in thumb movement amongst the three groups. However, there was one case of the delayed union in the ESIN-RPS group, five cases in the KW-R group, and three cases in the ESIN-A group. There was one case of non-union in the KW-R group. Skin irritation occurred in four patients of the ESIN-RPS group, which was significantly higher than the ESIN-A group (*P* < 0.05). The supination function of the ESIN-A group was worse than that of the other two groups. According to the Gartland-Werley standard of joint function, the excellent and good rates were 95.8% in the ESIN-RPS group, 86.5% in the ESIN-A group, and 86.1% in the KW-R group.

**Table 2:** Comparison of outcomes of radiographic and functional follow-up.

	ESIN-RPS	KW-R	ESIN-A	*P*_1_/*t*_1_ or *χ*^2^	*P*_2_/*t*_2_ or *χ*^2^	*P*_3_/*t*_3_ or *χ*^2^
Follow-up (month)	11.4 ± 2.6	38.6 ± 12.6	19.3 ± 9.9	0.001/10.197	0.001/3.741	0.001/7.785
Limited dorsiflexion	1	1	2	0.548/0.361	2.845/0.241	0.164/3.610
Delayed Union	1	5	3	0.104/4.521	0.145/2.979	0.019/7.087
Nonunion	0	1	0	/	/	/
Shin irrigation	4	5	1	0.339/0.913	0.049/3.864	0.211/0.565
Forearm rotation	0–90–180	0–90–180	15–90–180	/	/	/
Gartland-Werley criteria	Excellent: 23 cases, good: 1 case, fair: 1 case	Excellent: 25 cases, good:1 case,fair: 1 case	Excellent: 31 cases, good:3 case,fair: 2 case	0.108/2.576	0.110/2.557	0.003/0.945

Where *P* is the statistical probability, *t* is the statistical quantity, *P*_1_/*t*_1_ is ESIN-RP vs. KW-R, *P*_2_/*t*_2_ is ESIN-RP vs. ESIN-A, and *P*_3_/*t*_3_ is KW-R vs. ESIN-A. *χ*^2^ is the Fisher's exact test result.

### 3.4. Complications

During follow-up, two patients in the ESIN-A group had radial nerve injury. In the KW-R group, six cases had a superficial infection because of the k-wires exposed, and all had improved after several dressing changed. One patient in the ESIN-RPS group had a complete rupture of the tendon, and one patient in the KW-R group had a partial rupture of the tendon. After the K-wires pulled out, two patients had secondary fractures within 3 months. With regard to complications, there were significant differences between the ESIN-RPS and KW-R groups (*P* < 0.05), the ESIN-A and KW-R groups (*P* < 0.05), and the total incidence of complications in the KW-R group was significantly higher than that in the other two groups. Complications are summarized in Table 3.

**Table 3:** Comparison of the major complications in three groups.

	ESIN-RPS $\left(\bar{x}\pm s\right)$	KW-R $\left(\bar{x}\pm s\right)$	ESIN-A $\left(\bar{x}\pm s\right)$	*P*_1_/*t*_1_ or *χ*^2^	*P*_2_/*t*_2_ or *χ*^2^	*P*_3_/*t*_3_ or *χ*^2^
Mal-union	0	4	2	/	0.696/0.153	/
Nerve injury	0	0	2	/	/	/
Infection(superior/deep)	0	6	0	/	/	/
Tendon injury	1	1	0	0.522/0.361	/	/
Secondary fracture	0	2	0	/	/	/
Total	1	13	4	0.034/4.480	0.363/0.828	0.087/2.633

Where *P* is the statistical probability, *t* is the statistical quantity, *P*_1_/*t*_1_ is ESIN-RP vs. KW-R, *P*_2_/*t*_2_ is ESIN-RP vs. ESIN-A, and *P*_3_/*t*_3_ is KW-R vs. ESIN-A. *χ*^2^ is the Fisher's exact test result.

## 4. Discussion

DRMDJ fractures are located at a tendon-muscle transitional area with poorer healing ability and are more prone to delayed union or non-union compared to the metaphyseal or diaphyseal parts (8, 20). This study pioneered the use of ESIN-RPS fixation and compared this technique with previous techniques such as ESIN-A and KW-R. The outcomes of ESIN-RPS showed a greatly improvement of AP alignment quality and reduction of bleeding, radiation exposure, and operation times. It also increased the healing rate and accelerated the early rehabilitation exercise. Moreover, patients in this group were able to return to school earlier. Finally, this method reduced family and social costs, providing a reasonable choice for clinicians.

Compared with traditional ESIN-A and KW-R, ESIN-RPS has several advantages. Firstly, it avoids the physis injury of the distal radius, greatly reducing the possibility of premature physeal closure (PPC). Secondly, this procedure achieves better fracture alignment rate and reduces the incidence of delayed union and non-union. Thirdly, compared with ESIN-A, ESIN-RPS avoids the injury of radial nerve as well as supinator muscles, reducing the possibility of limited supination activity (7). Finally, the tail of the ESIN is placed on the surface of the deep fascia without direct contact with the extensor pollicis longus tendon, which minimizes the tendon rupture rate (15, 16). In this study, there was one case of complete tendon rupture in the ESIN-RPS group. The rupture site of the tendon was near the tip of the nail and was continuously wear until torn. The tendon was retracted by approximately 3 cm, and the tip of the nail was obliquely sharp. Tendon transplantation was performed using the palmaris longus muscle. The movement of the thumb was adequately restored at 4 weeks after the cast was removed.

The stability of a single intramedullary KW-R fixation is limited, and a well-placed trans-cortical KW-R fixation through the physis requires repeated insertion and multiple fluoroscopies, increasing the radiation exposure, tendon injury, and PPC (12, 17). In addition, the tail of the K-ware is exposed, which may cause infection. In this study, six patients had superficial infections. Furthermore, KW-R fixation has another disadvantage; a dilemma could occur when the K-wires should be removed while the fracture healing is delayed. Continuing fixation increases the risk of infection and affects joint activity. Conversely, if K-wires are removed, secondary fracture and non-union would happen, especially within 3 months after hardware removal (18–20). To reduce the risk of PPC, some researchers chose limited incision at the fracture site and anterograde insert the K-wires firstly, then performed retrograde trans-epiphyseal intramedullary K-wire fixation (21). However, a pin still is across the physis. Li et al. compared the results of the treatment of DRMDJ fractures with an external fixator and KW-R fixation, concluding that external fixator fixation had more advantages (10). However, external fixator related problems such as the inconvenience of upper limb movement and patient's fear should be considered.

To reduce external fixator-related problems or other complications, Han et al. explored the ESIN-A approach from the proximal Thompson incision. In this procedure, there is a risk of deep branch of radial nerve injury. In addition, the tip of the nail continuously wears the supinator muscle, resulting in limited supination (7). Furthermore, the removal of the ESIN caused supinator muscle injury again. In this study, two patients with limited thumb extension showed nerve degeneration which adjacent to the tail of the nail in the ESIN-A group. In an addition, half of the patients had instant loss of supination before the nail was removed and anxiety was spread amongst the patients.

Regarding the retrograde ESIN technique, some researchers used the elastic L-shaped prebending technique, and the results showed that more than half of the patients had >25% residual lateral displacement after surgery (6). To obtain a better alignment, Krohn et al. modified the method for an S-shaped prebending of a steel nail rather than an L-shaped type (22). The method is similar to that of ESIN-RPS, and it is difficult to achieve an elastic four-point support system due to the nature of rigid steel. Additionally, there is a lack of follow-up in that study; hence, the effectiveness of this technique is unclear. In this study, ESIN was used for precise shaping, and elasticity was used to achieve a stable support system. The results of the short-term follow-up and surgical comparison were summarized, which effectively complemented Krohn's study. With the development of orthopaedic materials, some scholars have used absorbable elastic materials for focal intramedullary fixation to treat DRMDJ fractures (23). Although the effect is better than that of K-wire fixation and does not require hardware removal, the materials are expensive, less stable, and may be broken during insertion (23).

This study had some limitations. Firstly, the sample size was small, especially in the ESIN-RPS group, which avianized the reliability of the statistical results. Secondly, this was a retrospective study conducted in various time periods, the operation was performed by different levels of surgeon. Thirdly, this study did not include secondary surgery or total costs, which led to information bias and reduced the advantages of KW-R fixation.

In conclusion, compared with ESIN-A and KW-R, ESIN-RPS has the advantages of shorter surgery time, less bleeding loss, less radiation exposure, better alignment, and fewer complications. The overall outcomes were better in the ESIN-RPS group than the ESIN-A and KW-R groups. Therefore, ESIN-RPS may be an effective choice for DRMDJ fractures.

## Data Availability

The raw data supporting the conclusions of this article will be made available by the authors, without undue reservation.
